# Bridging knowledge and practice in the prevention of child maltreatment in medicine: an analysis of counselling and training approaches

**DOI:** 10.3389/fpubh.2025.1588110

**Published:** 2025-07-31

**Authors:** Anna Eberhardt, Ulrike Hoffmann, Jörg M. Fegert, Oliver Berthold

**Affiliations:** ^1^Department of Child and Adolescent Psychiatry, Psychosomatics and Psychotherapy, Ulm University Hospital, Ulm, Germany; ^2^German Center for Mental Health (DZPG), Partner Site Ulm, Ulm, Germany; ^3^Child Abuse Outpatient Clinic, DRK Clinics Berlin, Berlin, Germany

**Keywords:** child maltreatment, counseling, training, healthcare professionals, medicine, prevention, violence

## Abstract

**Introduction:**

Protecting children from maltreatment is an important public health challenge. Despite increasing awareness, deficits exist in the training of healthcare professionals, who play a key role in the detection of maltreatment. Initiatives such as the medical child protection helpline and the online course “Child Protection in Medicine - a basic course for all healthcare professions” have been established in Germany to improve knowledge and competence among healthcare professionals. This study examines how collaboration between the two programs contributes to strengthening expertise and practice in child protection.

**Methods:**

Since 2016, the online course has offered flexible training for healthcare professionals on child protection. From 2017, the helpline has offered free 24/7 advice for healthcare professionals on suspected cases of child maltreatment. Both measures are evaluated about effectiveness and user satisfaction. The data analysis was conducted using descriptive analyses and *t* tests.

**Results:**

The helpline received a total of 4,911 calls between 2017 and 2024, mainly from physicians (61.7%) and psychotherapists (26.4%). The number of calls continuously increased. Most calls were made during working hours. The advice provided included assessments of maltreatment cases, legal issues, and referrals. The online course was used by 3,493 health professionals. Before the training, 90.5% of the participants stated that they needed more knowledge about child protection and 95.2% requested more flexible training. After the course, knowledge improved significantly, especially among unlicensed therapists (Cohen’s *d* = 1.8), medical students (*d* = 1.5) and nurses (*d* = 1.5). On the other hand, action competencies increased significantly across all professional groups. Of the participants, 40.3% were familiar with the helpline before the training.

**Discussion:**

This study finds that the helpline and the online course together strengthen professionals’ knowledge and action competencies in medical child protection. The high utilization of both services highlights existing knowledge gaps and the need for training. The increasing number of calls to the helpline underscores its relevance as a quick point of contact. The combination of individual counseling and structured training can improve child protection expertise comprehensively. An expansion of training and support services remains essential.

## 1 Introduction

Protecting children from abuse, neglect and other forms of maltreatment includes not only ethical but also social and health dimensions. The high prevalence of child maltreatment and neglect worldwide poses a serious threat to the well-being and development of children and adolescents (1–5). Child maltreatment and its often lifelong consequences are among the leading causes of health and social inequalities worldwide (6–8), which demonstrates the enormous importance of improving primary and secondary prevention of child maltreatment (child protection) in the public health sector. Despite increasing awareness and efforts at the political level, there is still a need to increase awareness and knowledge in the field of child protection, especially among those who are in direct contact with children, such as healthcare professionals (9). These professionals play a key role in the early detection of signs of child maltreatment and neglect, the implementation of protective measures and support for families in crisis situations due to their regular contact with children and families. The resulting relationship of trust and the possibility of identifying and treating maltreatment-related injuries and behavioral disorders allow healthcare professionals to act as critical interfaces between children, families, and protective services (10–12). In Germany, the Act on Cooperation and Information in Child Protection (KKG), which regulates collaboration between certain professional groups (including healthcare professionals) in cases of (suspected) child maltreatment, has been in force since 2012. This gives healthcare professionals a legally regulated authority to inform the youth welfare office, even without a release from the duty of confidentiality, if certain conditions are met (13). Effectively assessing and acting in child protection is complex and demands comprehensive knowledge of the causes, forms, and signs of child maltreatment, along with an understanding of relevant legal and ethical aspects. In addition, it requires insight into how other institutions function and how to work together with them, such as child protection services or law enforcement.

In recent years, advances in research and practice on medical child protection have led to a deeper understanding of the underlying risk factors and clearer guidance on how to proceed in cases of suspected child maltreatment (14, 15). However, a gap remains between the available knowledge and its application in clinical practice. Research shows that many healthcare professionals are confronted with cases involving child maltreatment but feel unsure about how to correctly interpret signs and take appropriate action (16–19). Furthermore, research shows large heterogeneity in professionals’ knowledge and application of legal frameworks regardless of whether they are subject to mandatory reporting laws (17). In addition, studies have shown that marginalized families are disproportionately often suspected of child maltreatment, including in the healthcare sector (20, 21). This can have serious consequences for the children concerned, who are dependent on the protection and intervention of medical staff (9, 22, 23).

The need to deepen and disseminate knowledge about child protection among healthcare professionals is therefore highly important to ensure comprehensive and effective care for children at risk. The Accreditation Council for Continuing Medical Education, in its Strategic Plan for 2022–2026, has advanced excellence in accredited continuing medical education/continuing professional development (CME/CPD) to leverage education to improve the quality of care for patients (24). Nevertheless, in Germany, gaps remain in current training structures and training regulations in medicine, and education on topics related to child protection is not sufficiently standardized (25). Further training courses are crucial to prepare healthcare professionals for these challenges and to ensure adequate support for those affected. In the field of medicine, however, there is often a lack of opportunity to obtain further training and new knowledge due to a severe shortage of time and personnel, which is likely to become even more acute in the coming decades (25–28). Adequate training can improve not only healthcare professionals’ confidence and safety when dealing with suspected cases of child maltreatment but also the overall quality and effectiveness of child protection measures (29, 30). Therefore, innovative approaches to knowledge transfer and dissemination among healthcare professionals are needed to ensure that current knowledge and best practices in child protection can be adequately implemented in the medical field. Technological developments allow us to focus on telecommunication and online approaches, which offer low-barrier access, the flexible and practical acquisition of knowledge, and various learning opportunities. This training is highlighted as an effective means of knowledge transfer in medicine, particularly in the field of child protection (31–33).

In this context, various initiatives have been developed in Germany to improve knowledge and practice in the field of medical child protection. Two key approaches are the medical child protection helpline (MCPH) and the online course “Child Protection in Medicine - a basic course for all healthcare professions” (OCPM). The MCPH, which has been available throughout Germany since 2017 and is funded by the German Federal Ministry for Family Affairs, Senior Citizens, Women and Youth, offers healthcare professionals the opportunity to obtain professional guidance in acute cases of (suspected) child maltreatment (https://kinderschutzhotline.de/). The OCPM was developed from 2015 to 2021 with funding from the German Federal Ministry of Health. Since 2021, it has been offered for a participation fee (https://kinderschutz-im-saarland.de/local/pages/view.php?id=6) on an e-learning platform funded by the Ministry of Labor, Social Affairs, Women and Health of the German state Saarland. The aim of the course is to raise awareness of child protection among healthcare professionals and to provide them with the necessary knowledge and tools to act effectively in their day-to-day work. The OCPM covers a variety of topics, from recognizing symptoms, conversation with partents of maltreatment to the legal basis for child protection in Germany.

**Figure 1:**
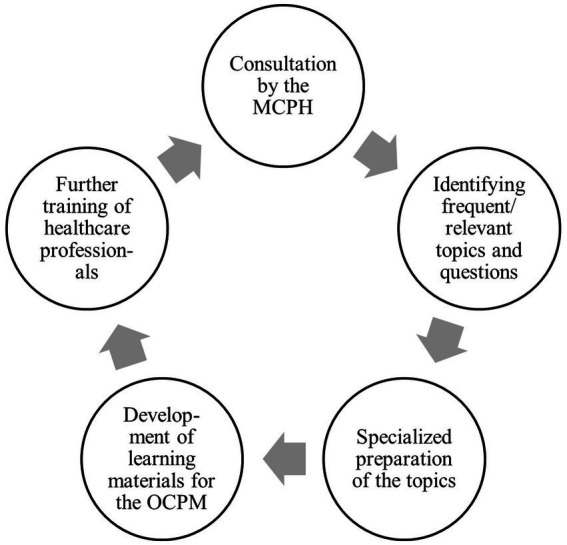
Feedback loop between frequent consultation requests from the medical child protection helpline (MCPH) and the online course “Child Protection in Medicine - a basic course for all healthcare professions” (OCPM).

The collaboration between the two groups fostered a distinctive synergistic effect. Questions that arose during consultation with the MCPH were systematically analyzed to identify frequent and relevant topics, which were then professionally prepared and translated into didactically suitable learning materials for the OCPM. The content developed in this way was incorporated into the training of healthcare professionals to improve their knowledge and skills in medical child protection. This can lead to new, more precise questions in practice, thereby establishing a continuous feedback loop between practical experience and scientifically founded knowledge transfer. This feedback loop was highlighted by the WHO’s regional status report on Europe in 2018 (34) (Figure 1).

It is important to examine whether this synergistic effect also helps to improve the knowledge and skills of healthcare professionals in their daily work. To improve the primary and secondary prevention of child maltreatment, this synergy must also contribute to improving healthcare professionals’ knowledge and confidence in their day-to-day work. This question is particularly important considering the high prevalence of child maltreatment (3, 35, 36) and the rate of missed cases in the healthcare sector (37, 38). An initial description of the collaboration was published in relation to abusive head trauma. Frequently occurring questions about this topic to the MCPH were prepared in a training unit for the OCPM and made available to a larger specialist audience (39).

In this study, we analyze anonymized consultation data from the MCPH to identify the challenges, and support needs healthcare professionals encounter in suspected cases of child maltreatment. These practical insights are compared to data from the OCPM, which assesses participants’ self-rated knowledge, action competencies and training needs in child protection before and after course completion. The aim of this study is therefore to analyze the data from the MCPH and the OCPM and to evaluate how these tools contribute to knowledge and competence building in medical child protection. We hypothesize firstly that the OCPM addresses key topics reflected in MCPH consultations and secondly that the combination of the two offers provides a good opportunity to increase the knowledge and action competencies of healthcare professionals, both in general and in relation to specific issues.

## 2 Materials and methods

### 2.1 Data collection

The MCPH and the OCPM are two central interventions in the field of counseling and training in child protection in Germany. Both conduct systematic data collection.

In 2017, the German federal ministry for families, senior citizens, women, and youth commissioned the University Hospital Ulm to establish a counseling service for healthcare professionals for suspected cases of child maltreatment. In cooperation with different partner sites, the MCPH was established as a service for healthcare professionals to receive guidance from specialized physicians on a 24/7 basis over the phone. The service is funded by the Ministry and is therefore free of charge for professionals who call the helpline. The consulting physicians are pediatricians, child and adolescent psychiatrists or forensic physicians. All the participants are certified child maltreatment physicians and can contact a senior specialist in all three subspecialties for consultations at any time. Healthcare professionals can receive guidance on any aspect of suspected child maltreatment and related issues. Counseling is anonymous and does not accompany the cases, which means that the counselors are not informed about the further course of a case. In Germany, accompanying counseling is offered by the youth welfare office. Since 2021, professionals from child and youth welfare institutions, including child protection services and civil courts dealing with out-of-home placement and custody issues, have used the service to resolve medical issues. The consultants document the calls after the end of the phone call in a secure online, semi-structured questionnaire. In addition to the date, time and duration of the call as well as the professional background and gender of the caller, this questionnaire also records the age group and gender of the child that the call was about. In addition, the topics discussed during the phone call were documented, as well as what advice was requested and how the advice was assessed at the end, respectively, where the greatest support was achieved. Both could be selected from a list and amended via a free text field.

The OCPM, which has been offered since 2016, also collects extensive data to evaluate training. Participants complete detailed surveys before and after taking part. In addition to information on demographics and professional background, questions on knowledge, training opportunities and training needs regarding child protection are addressed before working on the course. Before and after completing the course, the graduates’ knowledge and action competencies in child protection are assessed using self-assessment. The assessments are based on an end-point scale that was developed from evaluations of previous e-learning courses (40). For the analysis of knowledge, a score is calculated from eight items on epidemiology and the treatment of four types of maltreatment (physical maltreatment, emotional maltreatment, sexual violence, and neglect). The minimum score is 8, and the maximum is 48. Questions on other training courses on child protection and the dissemination of content and materials of the course to colleagues are asked after completing the course. The questionnaire for evaluating the MCPH and the OCPM can be found in Supplementary material 1.

### 2.2 Data analysis

The calculations in this study were conducted using the statistics and analysis software SPSS (version 29.0) (41). The calculations were intended to provide a comprehensive understanding of the available data. The mean values, standard deviations (SD) and percentages were given as characteristic values (42, 43). The self-assessment of knowledge and competencies in medical child protection was compared before and after the completion of the OCPM with the same items using a paired-samples t test. The effect size according to Cohen (d) was used for interpretation (44). The survey on the OCPM was approved by the ethics committee of the medical faculty at the University of Ulm on June 16, 2016. Because counseling is anonymous, this ethics committee ruled in January 2017 that no formal approval was required to conduct the survey about the MCPH.

## 3 Results

### 3.1 Guidance from the MCPH

The MCPH recorded a total of 4,911 documented calls from the healthcare sector during the survey period from July 1, 2017, to September 30, 2024. Eighty percent of the callers were female and almost two-thirds (61.7%) were physicians, followed by psychotherapists (26.4%) (Table 1).

**Table 1:** Sample of healthcare professionals calling the medical child protection helpline.

Gender	*n*	% of 4,562
Female	3,655	80.1
Male	907	19.9

Occupational group	*n*	% of 4,026
Physicians	2,483	61.7
Nursing staff	134	3.3
Psychotherapists	1,063	26.4
Nonlicensed therapists (e.g., occupational therapists, speech therapists)	146	3.6
Psychologists	109	2.7
Medical assistants	17	0.4
Emergency paramedics	74	1.8

**Figure 2:**
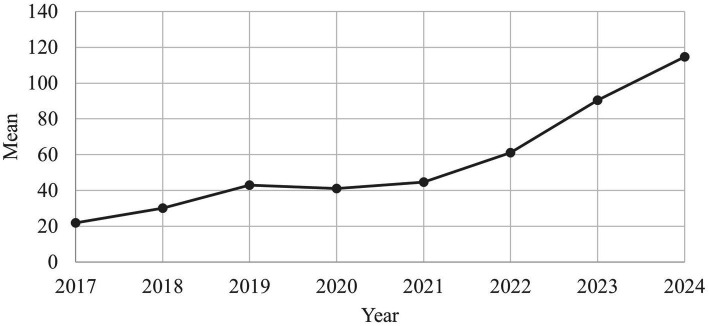
Average number of calls to the medical child protection helpline per month, 2017–2024 (*n* = 4,911).

Over the years, a continuous increase in the number of monthly calls has been observed (Figure 2). The advice is primarily aimed at physicians, nurses, and therapists, who are increasingly confronted with issues related to child maltreatment in their day-to-day work.

The data show that most calls to the MCPH concern children aged 13–15 years (*n* = 878; 17.9%), with a clear preponderance of female children in this age group (*n* = 634; 72.2%). Across all age groups, more calls were received for female children (*n* = 2,358; 56.51%) than for male children (*n* = 1,640; 39.30%) (Table 2).

**Table 2:** Distribution of gender and age among the children included in inquiries to the medical child protection helpline.

		Gender of the child							
		Female		Male		Gender unknown		Total	
		n	%	*n*	%	n	%	*n*	%
Age of the child [years]	Unborn	1	100.00	0	0.00	0	0.00	**1**	**100**
	<1	86	32.21	95	35.58	86	32.21	**267**	**100**
	1–3	222	46.35	214	44.68	43	8.98	**479**	**100**
	4–6	378	49.48	370	48.43	16	2.09	**764**	**100**
	7–9	279	45.66	321	52.54	11	1.80	**611**	**100**
	10–12	354	55.66	279	43.87	3	0.47	**636**	**100**
	13–15	634	72.21	237	26.99	7	0.80	**878**	**100**
	> 15	404	75.23	124	23.09	9	1.68	**537**	**100**
	**Total**	**2,358**	**56.51**	**1,640**	**39.30**	**175**	**4.19**	**4,173**	**100**

**Figure 3:**
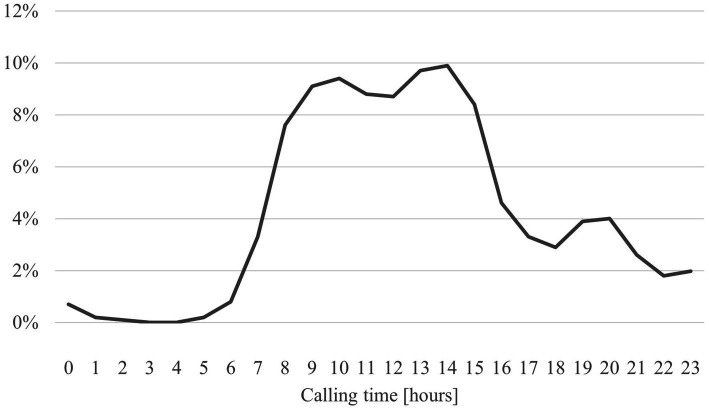
Time of calls to the medical child protection helpline in hours (*n* = 4,622).

Most of the calls were made during regular working hours. However, there was also a relevant proportion of calls outside core hours, particularly in the evenings (Figure 3). The average call duration was 13.2 min (S*D* = 8.7).

**Figure 4:**
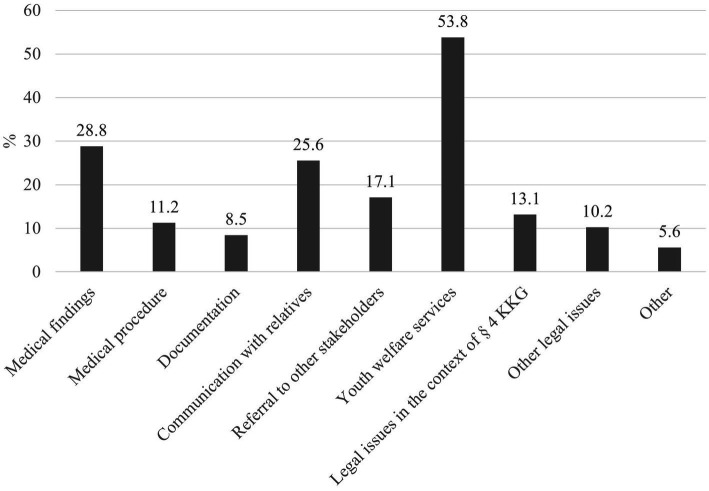
Content of the advice provided by the medical child protection helpline (multiple answers possible) (*n* = 4,911).

The topics discussed by callers varied. Many consultation requests concerned the assessment of medical findings for child maltreatment and possibilities of intervention. Also, possible referrals to support services such as youth welfare or how to conduct conversations in the context of child maltreatment were often discussed (Figure 4).

**Figure 5:**
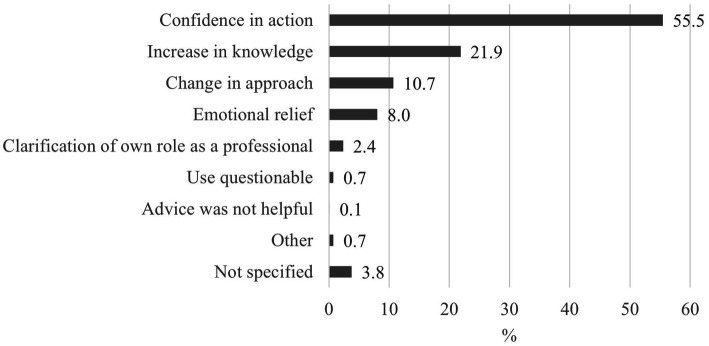
Assessment and advantages of advice from the medical child protection helpline (3,961).

Feedback from callers shows that in many cases, the advice not only helped to clarify acute issues but also strengthened the competencies of professionals. The benefits of the MCPH were frequently emphasized in cases of legal uncertainty, the assessment of suspicious circumstances and referrals to further support services (Figure 5).

### 3.2 Evaluation of the OCPM concerning growth in expertise and training opportunities for child protection in medicine

Of the 4,837 participants of the OCPM, 3,493 (72.2%) were healthcare professionals and were included in this analysis. Most of them were female (*n* = 2,962; 84.8%), 528 (15.1%) were male, and 3 (0.1%) indicated a diverse gender. On average, the participants were 39.6 years old (S*D* = 10.4) and had 10.9 years of professional experience (S*D* = 9.7). Most of the healthcare professionals were physicians (46.4%) (Table 3). Furthermore, 4,645 participants were asked if they have attended another training on child protection in addition to the online course, 1,642 (35.3%) affirmed this.

**Table 3:** Sample of healthcare professionals participating in the online course “Child Protection in Medicine - a basic course for all healthcare professions” (*n* = 3,493).

		Mean	SD
Age (years)		39.6	10.4
Professional experience (years)		10.9	9.7
		*N*	% of 3,493
Gender	Female	2,962	84.8
	Male	528	15.1
	Divers	3	0.1
Occupational group	Physicians	1,621	46.4
	Nursing staff	588	16.8
	Psychotherapists	473	13.5
	Nonlicensed therapists (e.g., occupational therapists, speech therapists)	390	11.2
	Medical students	116	3.3
	Psychotherapists in training	305	8.7

SD, standard deviation.

The results of the self-assessment of knowledge and competencies showed that licensed professionals, such as physicians and psychotherapists, as well as psychotherapists in training tended to exhibit a higher level of knowledge and competency than other professional groups did. Students and nonlicensed therapists scored significantly lower in both areas. The average level of knowledge in almost all professional groups increased significantly after completion of the online course. The greatest effects were recorded for unlicensed therapists (Cohen’s *d* = 1.8), followed by medical students (*d* = 1.5) and nursing staff (*d* = 1.5). Despite significant improvements, medical students had the lowest initial score and remained at a lower level after training than the other groups. In contrast, action competencies improved significantly in all professional groups, with the greatest effects observed among nonlicensed therapists (Cohen’s *d* = 1.6) and medical students (*d* = 1.8). The effect sizes were consistently high (Cohen’s *d* ≥ 1.1), indicating substantial learning gains in action competence as a result of the course (Table 4).

**Table 4:** Self-assessment of knowledge and action competencies in the field of child protection before and after completing the online course “Child Protection in Medicine - a basic course for all healthcare professions” (OCPM) among healthcare professionals by occupational group (paired-samples *t* test).

Occupational group	Knowledge (Min:8; Max:48)						Action competencies (Min: 1; Max:6)					
	T1		T2		Cohen’s effect size	*p*	T1		T2		Cohen’s effect size	*p*
	Mean	SD	Mean	SD			Mean	SD	Mean	SD		
Physicians (*n* = 1,569)	26.1	6.5	35.3	5.5	1.4	<0.001***	3.4	0.9	4.4	0.7	1.1	<0.001***
Nursing staff (*n* = 547)	23.9	7.2	36.0	5.8	1.5	<0.001***	3.1	0.9	4.4	0.8	1.2	<0.001***
Psychotherapists (*n* = 451)	28.2	6.7	37.3	4.9	1.3	<0.001***	3.5	0.9	4.6	0.7	1.2	<0.001***
Nonlicensed therapists (e.g., occupational therapists, speech therapists) (*n* = 390)	21.8	7.0	36.5	5.1	1.8	0.013*	2.8	0.9	4.4	0.7	1.6	<0.001***
Medical students (*n* = 100)	20.6	6.7	35.2	5.8	1.6	0.363	2.5	0.9	4.2	0.7	1.8	0.003**
Psychotherapists in training (*n* = 303)	26.5	6.8	36.9	4.7	1.5	<0.001***	3.3	0.9	4.5	0.7	1.4	<0.001***

*Significant at the 5% level; **Significant at the 1% level; *** Significant at the 0.1% level; T1, survey before starting the OCPM; T2, survey after completing the OCPM; SD, standard deviation.

Among the participants, 90.5% (*n* = 3,161) stated that they participated in the OCPM because they needed more knowledge on medical child protection for their daily work, and almost half of the participants (*n* = 1,670; 47.8%) reported that the OCPM was the only way for them to achieve this. Consequently, 95.2% (*n* = 3,326) of the participants stated the need for increased and more flexible training opportunities for child protection in medicine.

More than half of the healthcare professionals surveyed (58.7%; *n* = 2,052) shared the content and/or materials of the course with colleagues. A total of 38.7% (*n* = 1,352) shared the content verbally with colleagues, 2.7% (*n* = 93) used materials from the OCPM, and 15.5% (*n* = 543) disseminated the materials and shared content verbally. The majority (*n* = 1,846; 52.9% of 2,052) of those who disseminated information cited a lack of knowledge and a need for further training among colleagues on the subject as the reason.

After the MCPH was established in 2017, the OCPM participants were asked whether they were familiar with the MCPH prior to participation. A total of 40.3% stated that they were aware of the MCPH.

## 4 Discussion

This study analyzes data from two counseling and training approaches, the MCPH and the OCPM, in Germany and evaluates how a combination of these tools can be used to assess necessary areas of knowledge and skills, communicate this information and build competence in child protection among healthcare professionals. The results provide valuable insight into the existing knowledge and competence gaps in medical child protection. During the study period, a total of 8,724 calls (78% from the healthcare sector) and 3,493 participants in the OCPM were recorded. This corresponds with the lack of knowledge and confidence among healthcare professionals recognized by earlier studies (17, 19).

The clear majority of female participants in both the MCPH and the OCPM reflect the gender distribution in many healthcare professions, particularly in the fields of nursing and psychotherapy, where women are traditionally overrepresented (45). Most users of the MCPH and the OCPM were physicians (61.7%; 46.4%), which indicates their central role in the healthcare system, particularly in child protection work. Psychotherapists also constituted a significant group within MCPH and OCPM. In the OCPM, nurses and nonlicensed therapists were also relevant groups, which illustrates the importance of a multidisciplinary approach to child protection in the medical context. This has also been confirmed by other studies and is likely to increase healthcare professionals’ detection and confidence when dealing with (suspected) maltreatment cases (46–48). In many health professions, the topic of child protection is not just a task for physicians; it affects all professional groups that work with children and adolescents.

The participants’ self-assessments of their knowledge and competencies in child protection in the OCPM showed significant differences between the individual professional groups. Compared with other professional groups, licensed professionals such as physicians and psychotherapists provided higher scores in the self-assessment of their specialist knowledge and competence to act. While physicians and psychotherapists appear to be better prepared to recognize and deal with (suspected) cases of child maltreatment, nonlicensed therapists and medical students have lower levels of knowledge and competence. The low values in the self-assessment of students and nonlicensed professionals indicate the need to integrate the topic of child protection more strongly into the CME/CPD of these groups, as observed in other studies (49–53). This need is further supported by the evaluation of the OCPM: 90.5% of participants stated that they attended the training due to their own need for knowledge in the field of medical child protection. In addition, almost half of the respondents stated that the OCPM was their only opportunity to train in this area, highlighting the existing gaps in medical training on child protection. But 35% also attended another training on child protection, which may explain why the levels of knowledge and competencies was already high at the beginning of the course. All professional groups showed a significant improvement in knowledge and competence in child protection in medicine, with the exception of medical students, for whom the increase in knowledge was not significant. The effect sizes (Cohen’s d) consistently indicated large effects (*d* ≥ 1.1), suggesting substantial improvements because of the training. The results of the study also show that many participants shared their newly acquired knowledge with colleagues. More than half of the respondents stated that they had disseminated OCPM content to their colleagues, which is an indication of the high practical relevance and relevance of the content conveyed. It is particularly striking that the majority of those who passed on information did so on the grounds that their colleagues lacked knowledge and training in child protection. These results highlight not only the informal exchange of knowledge but also the need to create structured training opportunities that reach all employees in healthcare facilities equally.

The increasing number of calls to the MCPH over the years indicates the relevance of the MCPH as a point of contact for healthcare professionals in the field of child protection and an increasing awareness and willingness to request support in cases of (suspected) child maltreatment. Most of the calls were made during regular working hours, which indicates use by working professionals. However, there was also a relevant proportion of calls outside core hours, particularly in the evenings, which indicates the need for a flexible and easily accessible service. The content of the calls varied and ranged from the assessment of (suspected) cases of maltreatment to legal issues and referral to further support services. These topics confirm the results of other studies on the complexity and multilayered nature of the challenges that healthcare professionals experience when identifying and dealing with child maltreatment and the need for comprehensive and interdisciplinary counseling approaches (9, 54).

The feedback from the callers suggests that contacting MCPH helped them address specific cases and that the increase in knowledge and changes to their original approach also contributed to improved competence in future cases. It was frequently emphasized that the advice was highly beneficial in cases of legal uncertainty, in the assessment of suspicious circumstances and in referrals to further support services. These findings confirm the role of the MCPH as important support for healthcare professionals who need quick and sound advice in complex and stressful situations. The MCPH is a valuable tool to support healthcare professionals in urgent and complicated situations where informed decisions must be made rapidly. An even more differentiated analysis of calls in the context of case type, demographics or abuse concerns should be realized in the future to determine where the MKSH has the greatest impact.

A comparison of the healthcare professionals who completed the OCPM with those who received advice through the MCPH revealed that both samples were similar in terms of gender distribution and professional background. The results of the two studies can therefore be compared and are found to be complementary. The evaluation of the OCPM identified deficits in healthcare professionals’ knowledge and skills in child protection that were improved by completion of the course. The analysis of the calls to the MCPH allowed us to identify gaps in knowledge and skills and the needs of the target group. Furthermore, the analysis of data from the MCPH on counseling topics provides valuable insight into the reasons why professionals feel that they lack the competence to act, suggesting that in addition to their individual use, the two services benefit from close cooperation. For future research, it would be crucial to systematically investigate the impact of the OCPM by comparing the need for advice depending on the level of training. Such analyses could show whether the OCPM changes the use of the MKSH or has an impact on the type of cases consulted. The World Health Organization has described the combination of individual, continuously available, case-related, collegial consultation through the MCPH and the OCPM, which didactically addresses practical questions from the MCPH and returns them to practice in a feedback loop, as a lighthouse example for the European region in the field of child protection (34). The positive feedback on counseling highlights the need to further expand access to these support services and to strengthen the role of the MCPH as a complementary resource to existing training services. The MCPH and the OCPM not only offer short-term assistance but also contribute to the further development of professional competencies in the long term.

### 4.1 Limitations and strengths

This study has several limitations. First, reliance on self-assessment surveys may introduce response bias because the participants may have over- or underestimated their knowledge and competencies. Furthermore, the absence of a control group makes it difficult to attribute improvements solely to the interventions. In addition, the collected data are highly context dependent and limited to the specific target groups of the two initiatives, which may limit the generalizability of the results. Finally, potential biases due to unrecorded influencing factors, such as the participants’ previous experience or the quality of the data input, may influence the interpretation of the results. However, this study leveraged data from two well-established and systematically evaluated interventions, the MCPH and OCPM, in the field of child protection in Germany. Both programs utilize comprehensive data collection processes to ensure high-quality information about the participants’ demographics, professional backgrounds, and self-assessed improvement in knowledge and competencies. These aspects strengthen the validity of the results and allow a differentiated view of the approaches.

### 4.2 Conclusion

The results of this study highlight the urgent need for flexible further training opportunities in the field of medical child protection. The high level of participation in the OCPM and the increasing use of the MCPH underscore the need to continuously train professionals in this area and provide them with the necessary resources to recognize child welfare risks at an early stage and act accordingly. The combination of structured training courses and supporting resources such as the MCPH suggests that healthcare professionals can strengthen their competencies to meet the complex challenges of child protection.

In summary, both the OCPM and the MCPH play central roles in supporting healthcare professionals in cases of (suspected) child maltreatment. To continue this development in the long term, further investment should be made in public relations work, training and the expansion of resources. Both the OCPM and the MCPH offer decisive contributions to early detection, prevention, and advice in cases of child maltreatment and remain indispensable building blocks in the German child protection system.

## Data Availability

The raw data supporting the conclusions of this article will be made available by the authors, without undue reservation.
