# Supplementary material for: Bridging knowledge and practice in the prevention of child maltreatment in medicine: an analysis of counselling and training approaches
Source: Front Public Health. 2025 Jul 31;13:1588110. doi: 10.3389/fpubh.2025.1588110 (PMC12350255; doi:10.3389/fpubh.2025.1588110)
Supplement: Supplementary file 1 [file Data_Sheet_1.pdf]

Supplementary material 1:

Questionnaire on evaluation of the counselling of the medical child protection helpline (MCPH) and the online course “Child Protection in Medicine - a basic course for all healthcare professions” (OCPM)

Questionnaire on evaluation of the counselling of the medical child protection helpline (MCPH)

1. Date of the start of the call
    - [dd:mm:yyyy] (Only numbers in this format can be entered)
2. Time of the start of the call
    - [hh:mm] (Only numbers in this format can be entered)
3. Time of the end of the call
    - [hh:mm] (Only numbers in this format can be entered)
4. The caller was informed that the case description must be anonymous and that responsibility for the case discussed remains with the caller.
    - Yes
5. Which target group of the MCPH does the call belong to?
    - Healthcare
    - Youth welfare
    - Family courts
6. Professional Group of the Caller
    - Physicians
    - Nursing staff
    - Psychotherapists
    - Nonlicensed therapists
    - (e.g., occupational therapists, speech therapists)
    - Psychologists
    - Medical assistants
    - Emergency paramedics
    - Other [Free text]
    - not specified

7. Gender of the Caller
   - male
   - female
   - not specified

8. How old is the child who is the subject of the consultation?
   - Unborn
   - <1 year
   - 1-3 years
   - 4-6 years
   - 7-9 years
   - 10-12 years
   - 13-15 years
   - 16 years and older
   - Not specified

9. Gender of the child who is the subject of the consultation?
   - male
   - female
   - unkown

10. What was the content of your consultation? (Multiple answers possible!)
    - Medical findings
    - Medical procedure
    - Documentation
    - Communication with relatives
    - Referral to other stakeholders
    - Youth welfare services
    - Legal issues in the context of § 4 KKG
    - Other legal issues
    - Not specified

11. How did the consultation specifically help the caller?
    - Confidence in action
    - Increase in knowledge
    - Change of procedure
    - Emotional relief
    - Clarification of own role as a professional
    - Use questionable
    - Advice was not helpful
    - Other
    - Not specified

Questionnaire on evaluation of the online course “Child Protection in Medicine - a basic course for all healthcare professions” (OCPM)

T1: surveyed prior to working on the OCPM

T2: surveyed after working on the OCPM

1. Gender (T1)
   - o male
   - o female
   - o divers
2. Year of birth (T1)
   - o [yyyy] (Only numbers in this format can be entered)
3. What is your occupational group? (T1)
   - o Physicians
   - o Nursing staff
   - o Psychotherapists
   - o Nonlicensed therapists (e.g. occupational therapists, speech therapists)
   - o Medical students
   - o Psychotherapists in training
   - o Other [Free text]
4. How many years of professional experience do you have since completing your highest level of education? (T1)
   - o [yyyy] (Only numbers in this format can be entered)
5. Do you know the medical child protection helpline? (T1)
   - o Yes
   - o No
6. Please indicate your agreement with the following statements. (T1)

	completely disagree					Completely agree
I am participating in this program because I need more knowledge in the area of child protection for my everyday work.	o	o	o	o	o	o
I am participating in this course because it is my only opportunity to learn about child protection in medicine.	o	o	o	o	o	o
There is a need for broader and more flexible access to training opportunities in the field of 'child protection in medicine'.	o	o	o	o	o	o

7. Please answer the following questions by rating your knowledge of various aspects of child maltreatment on a scale from very limited to very extensive. (T1 & T2)

	Very limited					Very extensive
Epidemiology (prevalence, causes, and consequences) of physical abuse of children and adolescents?	○	○	○	○	○	○
Diagnosis, recognition, and treatment of physical abuse of children and adolescents?	○	○	○	○	○	○
Epidemiology (prevalence, causes, and consequences) of emotional (psychological) abuse of children and adolescents?	○	○	○	○	○	○
Diagnosis, recognition, and treatment of emotional (psychological) abuse of children and adolescents?	○	○	○	○	○	○
Epidemiology (prevalence, causes, and consequences) of neglect in children and adolescents?	○	○	○	○	○	○
Diagnosis, recognition, and treatment of neglect in children and adolescents?	○	○	○	○	○	○
Epidemiology (prevalence, causes, and consequences) of sexual violence against children and adolescents?	○	○	○	○	○	○
Diagnosis, recognition, and treatment of sexual violence against children and adolescents?	○	○	○	○	○	○

8. Please answer the following questions by rating your action competencies dealing with cases of child maltreatment on a scale from very limited to very extensive. (T1 & T2)
   - ○ Very limited
   - ○
   - ○
   - ○
   - ○
   - ○ Very extensive

9. Have you shared content from the online course with colleagues, either verbally or in the form of materials? (T2)
   - Yes, I shared content verbally.
   - Yes, I shared the Materials of the online-course.
   - Yes, I disseminated the materials and shared content verbally.
   - No
10. Why did you share the content and materials from the online course with your colleagues? (Multiple answers possible) (T2)
   - My colleagues don't have the time to complete the course themselves.
   - My colleagues asked me directly to do so.
   - My managers asked me directly to do so.
   - My colleagues lack knowledge on the topic of child protection.
   - There is a need for further training on this topic among my colleagues.
   - I consider the content and materials suitable for teaching this topic.
   - Other [free text]
11. Have you already participated in other training courses on child protection, or are you currently participating in other training courses? (T2)
   - Yes
   - No, there were no offers available for me.
   - No, there were offers, but I did not take advantage of them.
   - No, I did not inquire about other offers.
